# Hendra and Nipah Virus Infection in Cultured Human Olfactory Epithelial Cells

**DOI:** 10.1128/mSphere.00252-17

**Published:** 2017-06-28

**Authors:** Viktoriya Borisevich, Mehmet Hakan Ozdener, Bilal Malik, Barry Rockx

**Affiliations:** a Department of Pathology, University of Texas Medical Branch, Galveston, Texas, USA; b Department of Microbiology and Immunology, University of Texas Medical Branch, Galveston, Texas, USA; c Monell Chemical Senses Center, Philadelphia, Pennsylvania, USA; d Department of Viroscience, Erasmus University Medical Centre, Rotterdam, The Netherlands; Icahn School of Medicine at Mount Sinai

**Keywords:** Henipavirus, neuroinvasion, olfactory epithelium

## Abstract

Henipaviruses are emerging zoonotic viruses and causative agents of encephalitis in humans. However, the mechanisms of entry into the central nervous system (CNS) in humans are not known. Here, we evaluated the possible role of olfactory epithelium in virus entry into the CNS. We characterized Hendra virus (HeV) and Nipah virus (NiV) infection of primary human olfactory epithelial cultures. We show that henipaviruses can infect mature olfactory sensory neurons. Henipaviruses replicated efficiently, resulting in cytopathic effect and limited induction of host responses. These results show that human olfactory epithelium is susceptible to infection with henipaviruses, suggesting that this could be a pathway for neuroinvasion in humans.

**IMPORTANCE** Henipaviruses are emerging zoonotic pathogens that can cause acute and severe respiratory and neurological disease in humans. The pathways by which henipaviruses enter the central nervous system (CNS) in humans are still unknown. The observation that human olfactory neurons are highly susceptible to infection with henipaviruses demonstrates that the olfactory epithelium can serve as a site of Henipavirus entry into the CNS.

## Abstract (precis)

Henipaviruses are emerging zoonotic pathogens that can cause acute and severe respiratory and neurological disease in humans. The pathways by which henipaviruses enter the central nervous system (CNS) in humans are still unknown. The observation that human olfactory neurons are highly susceptible to infection with henipaviruses demonstrates that the olfactory epithelium can serve as a site of Henipavirus entry into the CNS.

## TEXT

Hendra virus (HeV) and Nipah virus (NiV) are emerging zoonotic pathogens that belong to the family *Paramyxoviridae*, genus *Henipavirus* (1). Henipaviruses (HeV and NiV) can cause acute and severe respiratory and neurological disease in humans, with an average case fatality rate of 57% (1–4). Transmission of henipaviruses is thought to primarily occur through contact with infected animals (horses, swine), human-to-human transmission, and consumption of contaminated date palm sap (5–8).

The pathways by which henipaviruses enter the central nervous system (CNS) in humans are still unknown. We previously showed that following intranasal challenge of hamsters with NiV or HeV, infectious virus could be isolated from the frontal lobe, including the olfactory bulb, earlier and at higher titers than in tissues other than the CNS, suggesting involvement of the olfactory bulb in HeV and NiV pathogenesis (9). Several studies have shown that henipaviruses can enter the CNS of mice, hamsters, and swine via the olfactory route (10–12). These studies showed that henipaviruses can infect the olfactory epithelium in the nasal turbinates and that infected neurons extend through the cribriform plate and into the olfactory bulb. Currently, it is unknown whether this is also an important route of infection in humans, due to the limited data on virus tropism in human cases of Henipavirus infection. No histological changes were found in the olfactory bulbs of nine NiV patients. However, magnetic resonance imaging (MRI) of 31 patients infected with NiV showed involvement of the uncus of the temporal lobe in 30% of cases (13). The uncus is covered by part of the olfactory cortex, thus providing indirect evidence of virus entry through the nasal airways, along the olfactory bulb, and into the uncus. In the current study, we determined the ability of henipaviruses to infect primary cultures of human olfactory epithelial cells and characterize the host response.

In order to investigate whether henipaviruses can replicate in human olfactory epithelial cells (hOE), hOE cultures were infected with HeV (horse isolate, Brisbane 1994), NiV strain Malaysia (NIV-M; human isolate, Malaysia 1998), and NiV strain Bangladesh (NiV-B; human isolate, Bangladesh 2004), at high and low multiplicities of infection (MOIs) of 1 and 0.01, respectively. hOE were derived from healthy, adult subjects, as previously described (14, 15). These cultures have been used to characterize the cellular composition and molecular expression of hOE and their response to odorants *in vitro* (14–17). Experiments were performed in triplicate with hOE from two different donors. Virus samples were obtained at various time points after infection, and viral titers were determined in a 50% tissue infective culture dose (TCID_50_) assay, as previously described (18).

**FIG 1:**
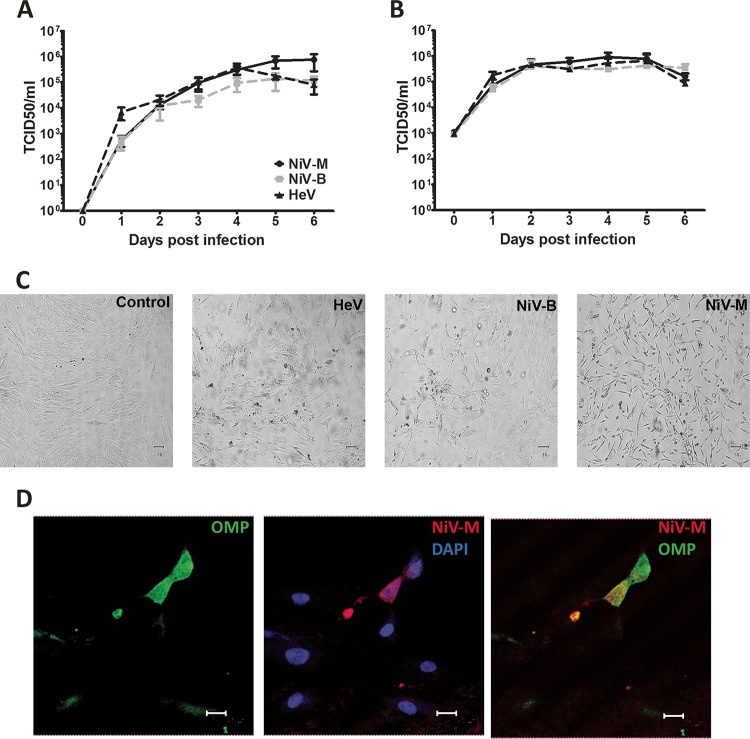
Henipavirus replication and tropism in human olfactory cultures. The kinetics of HeV (black dashed line), NiV-B (gray dashed line), and NiV-M (black solid line) replication in cultures of hOE infected at a low MOI of 0.01 (A) or a high MOI of 1 (B). Results are expressed as averages of 3 repetitions in hOE from 2 different donors; error bars represent standard errors of the means. (C) Cytopathic effect in control or HeV-, NiV-B-, or NiV-M-infected hOE cultures on day 5 p.i. (D) Representative panel of hOE cultures stained for immunofluorescent detection of OMP (green), NiV-M glycoprotein (red), and nucleus (4′,6-diamidino-2-phenylindole [DAPI], blue). In orange are hOE positive for both OMP and viral antigen. Bar, 25 µm.

All three henipaviruses replicated efficiently in hOE with no significant difference between the 3 strains used (Fig. 1A and B). At an MOI of 0.01, all three henipaviruses reached peak titers of 10^5^ to 10^6^ TCID_50_/ml by day 4 to 5 p.i. (Fig. 1A). At an MOI of 1, all three Henipavirus strains replicated to peak titers of 10^6^ TCID_50_/ml by day 2 p.i. (Fig. 1B). Infection of hOE resulted in a progressive cytopathic effect (CPE) as early as day 2 to 3 p.i. and was characterized by single syncytium formation by day 2 to 3, followed by extensive syncytium formation and cell death by day 5 to 6 (Fig. 1C). These data showed that henipaviruses can efficiently replicate in human olfactory epithelium.

To determine whether olfactory neurons were susceptible to infection with HeV, NiV-B and NiV-M, immunofluorescence staining of viral antigen (monoclonal antibody N-AH 1.3; detects NiV-B, NiV-M, and HeV glycoproteins) and olfactory marker protein (OMP; a marker for mature olfactory sensory neurons [OSNs]) was performed in Henipavirus-infected hOE. Viral antigen was only detected in OMP-positive cells, suggesting that all three Henipavirus strains exclusively infected OMP-positive OSNs in these cultures (Fig. 1D and data not shown). These data show that henipaviruses specifically target the OSNs in human olfactory epithelium.

**FIG 2:**
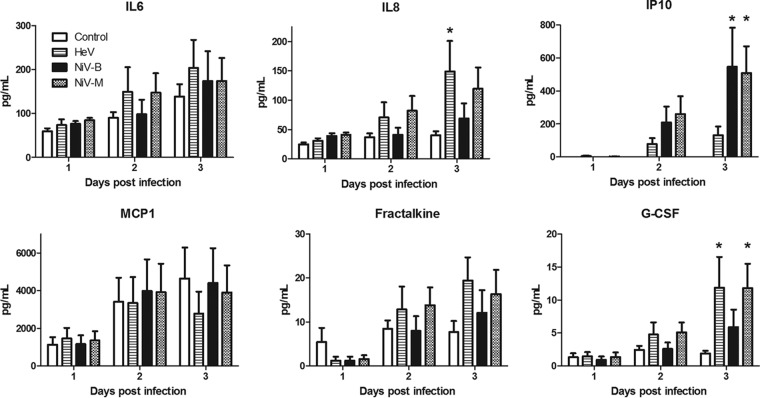
Cytokine levels in Henipavirus-infected hOE cultures. The concentrations of IL-6, IL-8, or IFN-γ-induced protein 10 (IP-10), MCP-1, fraktalkine, and G-CSF were determined in hOE cultures infected with NiV-M, NiV-B, or HeV at an MOI of 1. Concentrations are expressed as picograms of cytokine per milliliter of supernatant. The error bars represent the standard errors of the means. *, *P* < 0.05 (two-way ANOVA).

Finally, little is known about the ability of OSNs to produce cytokines and chemokines in response to viral infection. Instead, most studies focus on cytokine and chemokine responses in nasal washes, which include mediators produced by several different epithelial cell types in the nasal mucosa. To gain more insight into the immune response resulting from Henipavirus infection of the human olfactory epithelium, the levels of a panel of 15 chemokines and cytokines were quantified from hOE cultures infected at an MOI of 1 and sampled on various days postinfection (Fig. 2). The concentration of granulocyte colony-stimulating factor (G-CSF), granulocyte-macrophage colony-stimulating factor, alpha interferon (IFN-α), IFN-γ, interleukin 1α (IL-1α), IL-1β, IL-6, IL-8, IL-1 receptor agonist, chemokine ligand 10 (IP-10), eotaxin, monocyte chemotactic protein 1 (MCP-1), tumor necrosis factor alpha (TNF-α), fractalkine, and vascular endothelial growth factor A were quantified using a Milliplex human cytokine 15-plex immunoassay custom kit (Millipore).

Six mediators were detectable in the supernatant (Fig. 2), of which only the secretion levels of IL-8, IP-10, and G-CSF were significantly increased by one or multiple Henipavirus strains following infection of hOE, by day 3 p.i. IL-8 was significantly increased in hOE infected with HeV compared to controls, but not with NiV-B or NiV-M (*P* < 0.05; 2-way analysis of variance [ANOVA]). Interestingly, IP10 was only increased in hOE infected with either of the NiV strains but not with HeV. G-CSF responses were also increased in NiV-M- and HeV-infected hOE compared to controls. Increased levels of IL‑8 is associated with chronic inflammation of the nasal cavity (19). Previous reports on rhinosinusitis-associated olfactory loss have demonstrated that cytokines, particularly, TNF-α, IFN-γ, IL-6, nerve growth factor, and basic fibroblast growth factor play roles in cell damage, apoptosis, and loss of smell function (20, 21). However, these studies focused on the effects of cytokines on hOE proliferation and function and not on the production of cytokines by the hOE themselves. The exact roles of these mediators in Henipavirus pathogenesis remain unknown and require futures studies in animal models or patients. Overall, these data show that hOE have a limited immune response against HeV and NiV infection and that distinct HeV and NiV strains may differentially induce host responses in these cells.

In conclusion, our data show that human olfactory neurons are highly susceptible to infection with henipaviruses. This study demonstrates that the olfactory epithelium can serve as a site of Henipavirus entry into the CNS. We believe models like these allow for more detailed studies on the pathogenesis of Henipavirus infection in humans as well as other neurotropic viruses (22).
